# Arctic complexity: a case study on diel vertical migration of zooplankton

**DOI:** 10.1093/plankt/fbu059

**Published:** 2014-07-09

**Authors:** Jørgen Berge, Finlo Cottier, Øystein Varpe, Paul E. Renaud, Stig Falk-Petersen, Sawomir Kwasniewski, Colin Griffiths, Janne E. Søreide, Geir Johnsen, Anais Aubert, Oda Bjærke, Johanna Hovinen, Signe Jung-Madsen, Martha Tveit, Sanna Majaneva

**Affiliations:** 1 Faculty of Biosciences, Fisheries and Economics, University of Tromsø, 9037 Tromsø, Norway; 2 The University Centre in Svalbard, N-9171 Longyearbyen, Norway; 3 Scottish Association for Marine Science, Scottish Marine Institute, Oban, Argyll PA37 1QA, UK; 4 Akvaplan-Niva, Fram Centre for Climate and the Environment, N-9296 Tromsø, Norway; 5 Institute of Oceanology Polish Academy of Sciences, Powstancow Warszawy 55, 81-712 Sopot, Poland; 6 Department of Biology, Trondhjem Biological Station, Applied Underwater Robotics Laboratory, Norwegian University of Science & Technology (NTNU), N-7491 Trondheim, Norway; 7 Norwegian Polar Institute, Fram Centre for Climate and the Environment, N-9296 Tromsø, Norway; 8 Faculty of Biological and Environmental Sciences, University of Helsinki, FI-00014 Helsinki, Finland

**Keywords:** diel vertical migration, Arctic, ADCP, acoustics, zooplankton, physical forcing, predator–prey interactions, light regime, water masses, complexity

## Abstract

Diel vertical migration (DVM) of zooplankton is a global phenomenon, characteristic of both marine and limnic environments. At high latitudes, patterns of DVM have been documented, but rather little knowledge exists regarding which species perform this ecologically important behaviour. Also, in the Arctic, the vertically migrating components of the zooplankton community are usually regarded as a single sound scattering layer (SSL) performing synchronized patterns of migration directly controlled by ambient light. Here, we present evidence for hitherto unknown complexity of Arctic marine systems, where zooplankton form multiple aggregations through the water column seen via acoustics as distinct SSLs. We show that while the initiation of DVM during the autumnal equinox is light mediated, the vertical positioning of the migrants during day is linked more to the thermal characteristics of water masses than to irradiance. During night, phytoplankton biomass is shown to be the most important factor determining the vertical positioning of all migrating taxa. Further, we develop a novel way of representing acoustic data in the form of a Sound Image (SI) that enables a direct comparison of the relative importance of each potential scatterer based upon the theoretical contribution of their backscatter. Based on our comparison of locations with contrasting hydrography, we conclude that a continued warming of the Arctic is likely to result in more complex ecotones across the Arctic marine system.

## INTRODUCTION

Diel vertical migration (DVM) of zooplankton is a characteristic feature of the world's oceans and lakes, and is suggested to be the largest synchronized movement of biomass on the planet (Hays, 2003). Since the phenomenon was first described almost two centuries ago, there have been numerous studies into both the adaptive significance of this behaviour and its ecosystem consequences (for two reviews, see Hays, 2003; Ringelberg, 2010). Research on DVM has focused both on the proximate and the ultimate explanations of the behaviour (e.g. Hays *et al*., 2001; Lampert, 1989; Ohman, 1990), as well as on the implications for the ecosystem regarding trophic interactions and the biological carbon pump (e.g. Buesseler *et al*., 2007).

In the Arctic, the phenomenon of DVM is rather poorly understood, yet it is likely to have an important role to play in terms of the fate of carbon sequestration, food web interactions and the coupling processes between the pelagic and benthic realms. In general, zooplankton performs vertical migrations to balance the need to feed close to the surface where food is generally most abundant, but also to reduce the accompanying risk of being eaten in these shallow waters (e.g. Hays, 2003) where illumination creates favourable conditions for visual predators (Gliwicz, 1986). In the high Arctic, with its unique light regime, this behaviour has been frequently observed during autumn and spring when the day–night cycle is pronounced (e.g. Blachowiak-Samolyk *et al*., 2006; Cottier *et al*., 2006; Falk-Petersen *et al*., 2008; Wallace *et al*., 2010), whereas a number of studies have failed to find any coordinated vertical migration during periods of continuous light (e.g. Blachowiak-Samolyk *et al*., 2006; Fisher and Visbeck, 1993; Kosobokova, 1978). Recently, Berge *et al*. (Berge *et al*., 2009, 2012a) demonstrated that active DVM is performed by multiple species of zooplankton even during the polar night at a time of year when the ambient light is generally assumed to be insufficient to cue their ascent/descent. Wallace *et al.* (Wallace *et al.*, 2010) identified six separate phases of DVM throughout the year, each of which had a distinct pattern, suggesting that different species were dominating the observed patterns during different seasons. However, our current state of knowledge does not yet permit any reliable identification of the species responsible for this behaviour, nor the mechanisms regulating the vertical positioning of the organisms. Both of these factors are critical for a holistic understanding of the patterns and processes governing Arctic marine systems, and are major obstacles for any predictive understanding of how ecosystem processes are likely be affected by the current warming of the Arctic Ocean and surrounding shelf seas.

Most DVM studies from the Arctic (but see, e.g. Cisewski *et al*., 2010; Lavery *et al*., 2007 for examples in other regions of the world) have been carried out using either echo sounders (Falk-Petersen and Hopkins, 1981; Falk-Petersen *et al*., 2008; Rabindranath *et al*., 2011), ADCPs (Berge *et al*., 2009; Cottier *et al*., 2006; Fisher and Visbeck, 1993; Wallace *et al*., 2010) or zooplankton nets (e.g. Blachowiak-Samolyk *et al*., 2006; Longhurst *et al*., 1984), with the robustness of the observations and conclusions limited by the inherent bias and limitations of each of the sampling methods. Many of these studies have been opportunistic with interpretation of the DVM signal being based on incomplete or non-coincident data series. From the Arctic, partly for logistical reasons associated with sampling during the long polar night, most studies have adopted an approach involving moored ADCPs. There is a long track record of using ADCPs to study DVM patterns both in the Arctic and elsewhere (e.g. Cisewski *et al*., 2010; Cottier *et al*., 2006; Tarling *et al*., 2002; Wallace *et al*., 2010 for some recent examples) with the acoustic signal converted to a volume backscatter. However, this use of an ADCP is not ideal as the instruments are generally not calibrated as would be a standard echo sounder. However, and despite the concerns regarding aspects of the data provided by the instruments, they are widely used both in moored operations as well as fitted onto moving vessels and AUVs (Berge *et al*., 2012a; Schofield *et al*., 2010). Acoustic instruments moored over the entire annual cycle can provide us with a unique temporal appreciation of DVM patterns. Such a seasonal perspective is essential to achieve a more complete and quantitative understanding of pelagic community dynamics as well as the role of DVM in zooplankton annual routines (Varpe, 2012).

Although important for our understanding of the pelagic system, methods using ADCPs typically emphasize the broader migratory picture rather than the detailed characteristics of the signal (Wallace *et al*., 2010). Accordingly, and in line with a traditional view of short, low-diversity food chains in Polar Regions (Smetacek and Nicol, 2005), patterns of DVM in the Arctic are generally conceived as a single uniform layer of zooplankton, visible in the acoustic data as a Sound Scattering Layer (SSL), migrating in one synchronized response. While we know from other systems that the vertically migrating components of the pelagic community can often be seen as distinct SSLs (e.g. Klevjer *et al*., 2012 from the Red Sea), such information and understanding is to a large part missing from Arctic systems. Yet, given the importance of current changes in primary productivity (Arrigo *et al*., 2012) and sea ice extent (Cavalieri and Parkinson, 2012), there is an imminent requirement to understand the processes regulating vertical migration of zooplankton in the Arctic. This is particularly important if we want to properly account for its contribution to carbon cycling (Wallace *et al*., 2013) through coupling processes between the sympagic, pelagic and benthic systems.

Here, we address the nature of Arctic DVM patterns in terms of their complexity, species composition and drivers (biotic and abiotic). We present the results from a study of DVM in the Arctic collected from four contrasting sites around the high Arctic archipelago of Svalbard (78–80°N) during the autumn equinox, combining net-based zooplankton collection with moored acoustic instruments at a time of strong diel change in illumination. Based on this, we address our two main research questions. First, to what extent is there uniformity in the patterns of DVM occurring in arctic locations with contrasting hydrography? Second, can variations in DVM patterns be related to the physical environment? When observing DVM with acoustics, it is inevitable that we are also interested in the species composition of any SSL. Therefore, net samples were used both to detect organisms present at different depth layers in the water column and as a basis for calculating the theoretical backscatter of organisms that *de facto* are present in the water column. Understanding the identity of the migrants provides important insights into cues for vertical migration and the potential implications of this behaviour for carbon retention and export. It is also a vital step towards a more comprehensive understanding of the patterns and processes governing Arctic marine systems in an era of strong climatic change.

## METHOD

### Study area

**Fig. 1.**
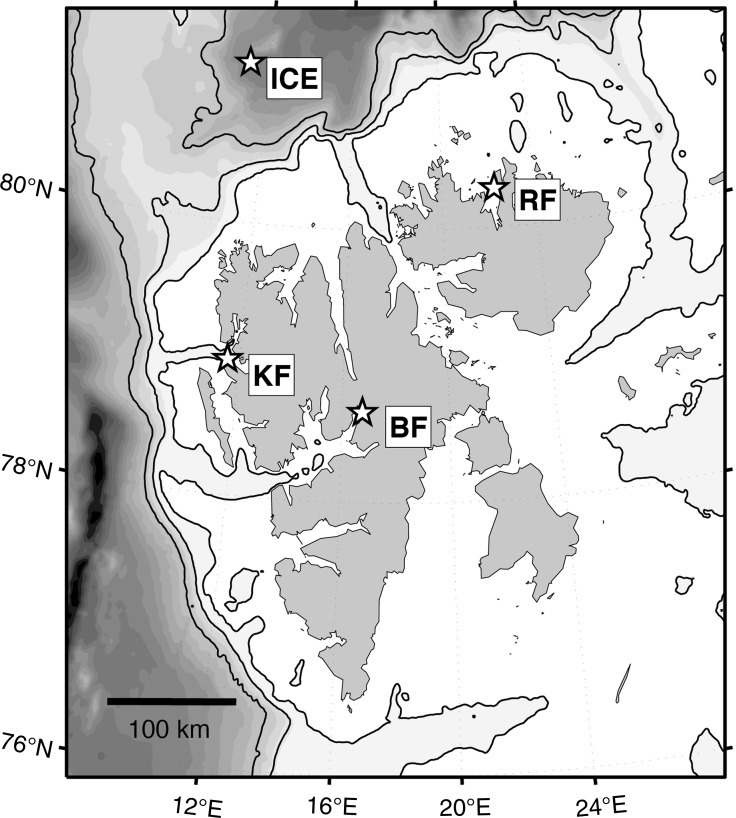
Regional map of the study area with stars indicating the four sampling sites and labelled Kongsfjorden (KF), Rijpfjorden (RF), Billefjorden (BF) and Ice Station (ICE). Bathymetric contours are at 200, 500 and 1000 m with the relief enhanced by shading.

The study was carried out from R/V *Helmer Hanssen* between 16th and 28th of September 2010. The period for observations was centred on the equinox to ensure the maximum diel cycle in solar irradiance. Data were collected at four sites in the Svalbard archipelago: Kongsfjorden, Rijpfjorden, Billefjorden and an Ice Station north of Spitsbergen (Fig. 1 and Table I). Kongsfjorden on the western coast of Spitsbergen is periodically dominated by intrusions of warm and saline Atlantic Water (AW) from the West Spitsbergen Current (Cottier *et al*., 2005; Svendsen *et al*., 2002) and has been largely free of sea ice during the past six winters. It displays many sub-arctic characteristics in terms of its water properties and this is reflected in the zooplankton species composition (Kwasniewski *et al*., 2003; Willis *et al*., 2006). Rijpfjorden at the northern part of Nordaustlandet is predominantly influenced by Arctic Water (ArW) (Ambrose *et al*., 2006; Søreide *et al*., 2010) and, in contrast to Kongsfjorden, is covered by sea ice for 6–8 months of the year. Billefjorden is a silled fjord in the inner part of the Isfjorden complex on the western coast of Spitsbergen. It is ice-covered for most of the winter and spring each year (Nilsen *et al*., 2008), and experiences rather little influx of AW (Arnkværn *et al*., 2005; Nilsen *et al*., 2008). The Ice Station north of Spitsbergen was located over deep water (>2000 m) and within the Marginal Ice Zone with a very dense cover of drifting sea ice. In our later analysis, water masses are defined according to Cottier *et al*. (Cottier *et al*., 2005); AW with T > 3.0°C and S > 34.65, ArW with T < 1.0°C and 34.30 < S < 34.80, surface water (SW) with T > 1.0°C and S < 34.00 and Winter Cooled Water (WCW) having T < −0.5°C and S > 34.4.

**Table I:** (A) Mooring position, depth and deployment time at each site, and the depth layers for MPS (multinet plankton sampler) and MIK (Method Isaac Kidd)/WP3 net samples at the four sites. (B) Acoustic Doppler current profiler (ADCP) mooring arrangement giving the instruments depths, orientation and profiling range.

Site	Position	Depth (m)	Mooring deployment and recovery dates/times	MPS depths (m)	MIK/WP3 depths (m)
(A)					
Kongsfjorden	78°59.58N	315	16.09.10 20:00	300–200, 200–100, 100–50, 50–20, 20–0	100–0 m (MIK)
	11°32.70E		18.09.10 07:00		
Rijpfjorden	80°17.07N	212	19.09.10 05:30	215–100, 100–50, 50–20, 20–0	50–0 m (MIK)
	22°16.08E		20.09.10 16:30		
Billefjorden	78°39.79N	186	27.09.10 06:30	150–100, 100–50, 50–20, 20–0	50–0 m (MIK)
	16°45.42E		28.09.10 20:30		
Ice Station	81°10N	2000	23.09.10 14:00	350–200, 200–100, 100–50, 50–20, 20–0	50–0 m (WP3)
	11°05E		25.09.10 02:30		
(B)					
Site	Instrumentation	Depth (m)	Orientation	# Data Bins	Depth range (m)
Kongsfjorden	ADCP 1	96	Upward	21	90–10
	ADCP 2	191	Upward	24	185–93
	ADCP 3	194	Downward	30	200–316
	CTD	31.5	–	–	–
Rijpfjorden	ADCP 1	83.5	Upward	18	77–9
	ADCP 2	175.5	Upward	24	169.5–77.5
	CTD	17	–	–	–
Billefjorden	ADCP 1	79	Upward	17	73–9
	ADCP 2	82	Downward	30	204–88
	CTD	28.5	–	–	–
Ice Station	ADCP 1	6.5	Downward	20	88.5–12.5
	ADCP 2	162.5	Upward	18	156.5–88.5
	ADCP 3	164	Downward	30	286–170
	CTD	31.5	–	–	–

The CTD was fitted with a PAR sensor. The same instruments and sensors were used at all stations.

In order for sampling to coincide with the anticipated close coupling between the timing of vertical migrations and the timing of the daily cycle of irradiance (Cottier *et al*., 2006; Tarling *et al*., 2002), all net sampling was conducted at a time corresponding to the local sun noon (LSN) (when the sun is at its zenith) and the local midnight (LM). LSN and LM were determined for each site by their longitude with the time of LSN/LM changing by 4 min for each degree of longitude. The longitude range in this study spans 11 degrees (see mooring positions in Table IA) equating to a difference in 44 min in the timing of LSN/LM between the most westerly (Ice Station) and most easterly (Rijpfjorden) sites, despite all sites being within the same time zone. To aid comparisons between sites, all data have been plotted onto a time axis based on the local time for each site, and all the times reported throughout the paper are referenced to these local times.

### Acoustics and physical parameters

At each site, an instrumented mooring was deployed for a minimum of 36 h, thereby ensuring that the nets and acoustics covered at least one full DVM cycle. The moorings in the shallower fjord sites were anchored to the seabed and retrieved using an acoustic release. At the deeper Ice Station, the mooring was suspended from the ship, which then followed the path of the ice floe to which it was anchored; drifting ∼4.5 nautical miles during the 2-day sampling period. At each site, a Seabird Electronics 911 CTD unit was used to obtain vertical profiles of temperature and salinity. The unit was fitted with a calibrated Seapoint fluorometer to provide Chl *a* (μg L^−1^) profiles as a measure of phytoplankton biomass, measured as emitted fluorescence at 685 nm. Additionally, a Seabird Electronics 19+ CTD unit fitted with a Biospherical Instruments scalar irradiance sensor, *E*_o_ (400–700 nm, photosynthetic active radiance, PAR, µmol photons m^−2^ s^−1^), was placed on the moorings.

**Fig. 2.**
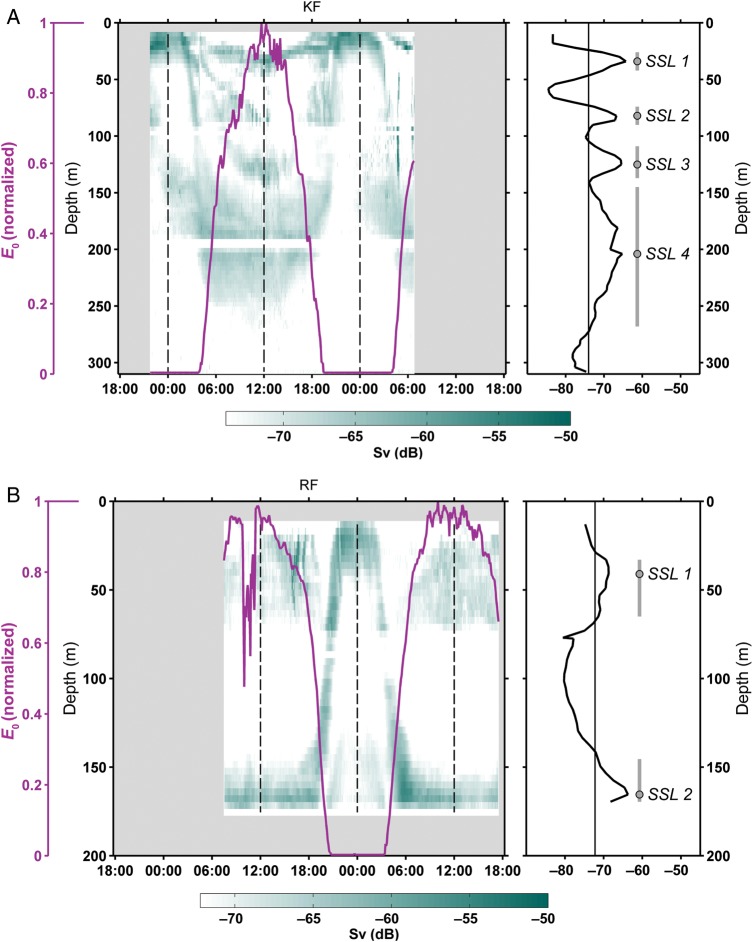

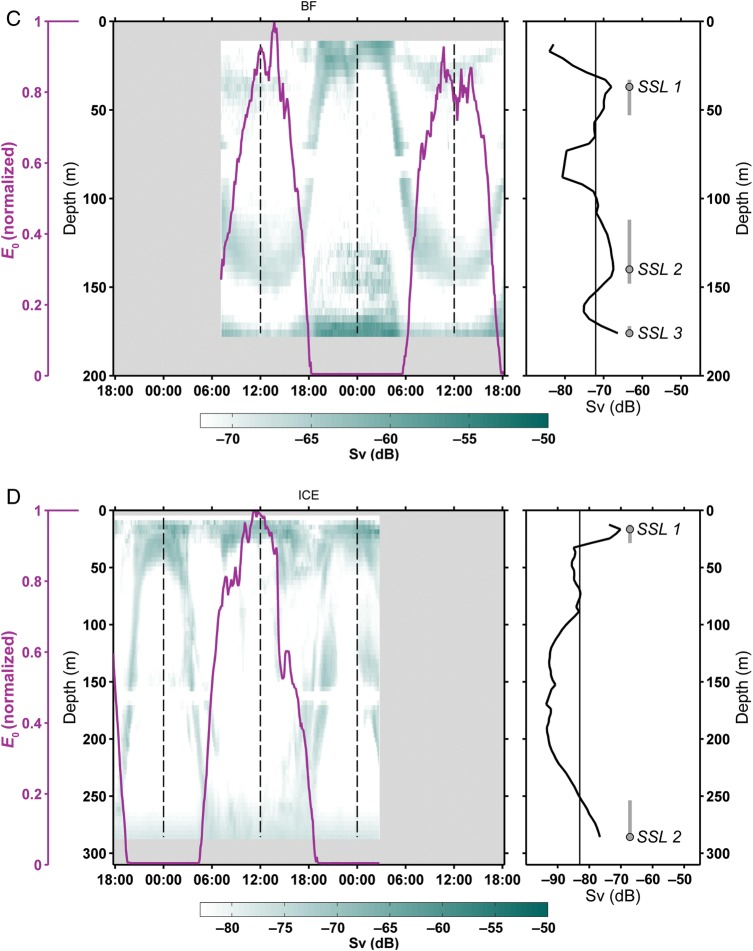
Distribution of the absolute backscatter for values greater than the mean Sv value (in dB) observed over a 36 h period at each of the four sampling sites: (**A**) Kongsfjorden, (**B**) Rijpfjorden, (**C**) Billefjorden and (**D**) Ice Station. The green colour bar ranges from white (the mean Sv value over the deployment period) to dark green (the maximum Sv value measure over the deployment period). The full backscatter distribution is given in Supplementary data, Fig. S1. LM and LSN are indicated by vertical dashed lines. Ambient solar irradiance (*E*_0_) is overlain in purple and normalized to the maximum measured value at each site. The detection of the SSLs at LSN is illustrated in panels to the right of each subfigure. The vertical grey line indicates the mean Sv value over the 36 h period, with the Sv profile at LSN overlain (black line). The SSLs are defined as those regions of enhanced Sv (relative to the deployment mean value) and depicted by thick grey vertical lines with the grey dot indicating the depth of peak Sv in each SSL.

Each mooring was fitted with 307.2 kHz RDI Acoustic Doppler Current Profilers (ADCP) (Table IA and B). As each ADCP is able to sample over a range of about 100 m, two ADCPs were deployed in Rijpfjorden (212 m) and Billefjorden (186 m), and three in Kongsfjorden (315 m) and at the Ice Station (2000 m). Full water column coverage was limited by the inability of the ADCPs to record data within the blanking distance of 4 m from the transducer head and within 10 m of the surface. The ADCPs were configured to measure the mean echo strength from contiguous bins of 4 m and from ensembles of 60 acoustic pings at a rate of one ping per second. The echo-strength data was then converted to a measure of the absolute backscatter (Sv, dB), following the application of the SONAR equation outlined in Deines (Deines, 1999, but see also Cottier *et al*., 2006, and references therein). The absolute backscatter over a 36-h period at each site is presented in Fig. 2 and Supplementary data, Fig. S1. Swimming speeds of the constituent zooplankton are inferred from the inclination of the SSLs (Cottier *et al*, 2006).

We note that the transducers on the ADCPs are not calibrated using standard target techniques (Foote *et al*, 1987) and hence do not provide an accuracy comparable with echosounder techniques. This is because backscatter strength is not always directly related to the size of a target or the abundance of targets, as physiological differences in shape, tissue type and orientation will affect the target strength of scatterers (e.g. Stanton *et al*., 1994, 1998a,b and references therein). However, careful application of the methodology following Deines (Deines, 1999) has been shown to give good correspondence to echo sounder techniques (Brierley *et al*, 2006). Further, organisms that contain a swimming bladder such as fish will give a much higher target strength and scattering value than the zooplankters. Accordingly, one would expect that any pelagic fish that enters the acoustic beams would give rise to a strong backscatter signal. However, such strong backscatter signals from fish are routinely rejected by the ADCP internal data quality filters (RDI, 1996).

### Zooplankton

Zooplankton sampling took place close to the fixed moorings. Mesozooplankton were sampled with Multi Plankton Sampler (MPS, Hydro-Bios, Kiel, equipped with five closing nets, mesh size = 200 μm, opening = 0.25 m^2^). The sampling depths were standardized between sites and are presented in Table I. To capture any change in the vertical positioning of zooplankton at the extremes of the diel cycle of illumination, the MPS sampling was carried out during LSN and LM. Macrozooplankton (i.e. larger chaetognaths, krill, ctenophores, amphipods and pteropods) were immediately removed from the sample, measured (length, width) and counted, after which the samples were stored in borax buffered 4% formaldehyde-in-seawater solution and transported to land for later analysis. In the lab, the smaller remaining size fraction of zooplankton was identified to the species level. Sub-samples of 5 mL, obtained using a wide-mouthed pipette, were counted until 100 individuals of *Calanus* spp. from each sample were identified to stage and then measured according to Kwasniewski *et al*. (Kwasniewski *et al*., 2003). Only stages CIV and above were included in analyses, because these stages are by far the most dominant at this time of year at the selected sites (e.g. Daase *et al*., 2008), but also because the contribution to acoustic backscatter decreases with organism size.

Macrozooplankton was collected during one LSN and one LM with a MIK net (mesh size = 1.5 mm with a filtering cod end, opening = 3.15 m^2^) in the upper 50 m (100 m in Kongsfjorden). At the Ice Station, with a high concentration of sea ice, the larger MIK net was replaced by the smaller WP3 net (mesh size = 1000 μm, opening = 1 m^2^). For all nets, the counts were converted to individuals per unit volume (ind. m^−3^) and are presented in Table II. To minimize any influence of spatial heterogeneity of the sampled zooplankton community, sequential net hauls were taken over a maximum period of 1 h. Length and width of up to 100 random individuals in each taxonomic group at each site were measured in order to establish a size distribution, length–width ratios and standard deviations needed for target-strength calculations (see below).

**Table II:** Abundance (m^−3^) of the main zooplankton groups at LSN and LM and the theoretical backscatter (dB) in brackets calculated for all organisms collected using Eqn (2) and calculated TS values.

Taxon	Kongsfjorden		Billefjorden	
	LSN 0–100 m	LM 0–100 m	LSN 0–50 m	LM 0–50 m
(A)				
*Mertensia ovum*	0.127	0.283	0.994	0.636
Krill (small)	0.143 (−108)	1.312 (−99)	0.006 (−122)	0
Krill (large)	0	0	0.019 (−102)	2.878 (−80)
Chaetognaths	1.971 (−122)	1.134 (−108)	0.994 (−109)	12.891 (−97)
*Themisto abyssorum*	0.051 (−109)	1.054 (−95)	0	0
*Themisto libellula*	0	0	0.083 (−92)	0.968 (−81)
Other zooplankton	0.032	0.003		
*Calanus* spp.	74.3 (−83)	54.9 (−85)	14 (−91)	21.9 (−89)
(B)				
Taxon	Rijpfjorden		Ice Station	
	LSN 0–50 m	LM 0–50 m	LSN 0–50 m	LM 0–50 m
*Mertensia ovum*	3.885	1.032	0.02	0
Krill (large)	0	0.356 (−89)	0	0
Krill (small)	0.134 (−109)	0.153 (−108)	0.02 (−117)	0
Chaetognaths	0.115 (−118)	9.808 (−99)	0.18 (−116)	0.84 (−109)
*Themisto libellula*	0.229 (−87)	0.064 (−93)	0.18 (−88)	0
*Themisto abyssorum*	0	0.013 (−115)	0	0
*Limacina helicina*	0.057	0.013	0	0
*Onisimus glacialis*	0.006	0.013	0	0
Other zooplankton	0.051	0.102	0.02	0.04
*Calanus* spp.	15.5 (−90)	14.7 (−90)	3 (−97)	23.14 (−88)
*Paraeuchaeta glacialis*	0	0	0	0.28
Ostracods	0	0	0	0
Other zooplankton	0	0	0.02	0.04
*Apherusa glacialis*	0	0	0	0.6
*Metridia* sp.	0	0	1.0 (−102)	78.0 (−83)

(A) Kongsfjorden and Billefjorden, (B) Rijpfjorden and Ice Station.

### Identification of Sound Scattering Layers

For each site, the vertical distribution of acoustic SSLs at LSN was identified objectively in the backscatter data (Fig. 2). A mean vertical profile of backscatter at LSN was calculated from those data spanning a 1 h period; 30 min either side of the time of LSN. The SSLs were then defined to be the peaks in the backscatter profile where values exceeded the overall mean backscatter calculated from all data collected during the 36-h measurement period. The vertical extent of each SSL was also determined to be the range in the water column where the backscatter at LSN exceeded the overall mean backscatter. We also performed similar analysis for identifying SSLs at LM.

### Analyses of acoustic backscatter

Scattering models have been developed for three main types of scatterers (Stanton *et al*., 1998a,b); fluid-filled organisms such as krill, elastic-shelled organisms such as gastropods, and gas-bearing organisms such as siphonophores. To interpret the measured values of absolute backscatter, the target strength (TS, in units of dB) of each zooplankton species was calculated based on the randomly oriented fluid bent cylinder model (Stanton *et al*., 1994):

(1) $$\begin{array}{r} \text{TS}=10\;\log\{0.08R^{2}L^{2}\beta_{D}^{-1}[1-\text{exp}(-8\pi^{2}\;f^{2}D^{2}s^{2}c^{-2})\\ \;\;cos(\pi fDc^{-1}{\left(4-\frac{1}{2}\pi fDc^{-1}+0.4\right)}^{-1})]\} \end{array}$$

where *R* is the reflection coefficient = 0.058 (Greene *et al*., 1998), *L* the mean body length (m), *s* the (standard deviation of *L*)/*L*, *D* the mean body width (m), *β_D_* = *L*/*D*, *f* the acoustic frequency in Hz = 307.2 × 10^3^, and *c* the speed of sound in water, defined here as 1500 m s^−1^. The theoretical backscatter (Sv, dB) of each taxonomic group of zooplankton present in the water column is then estimated by (see also Cottier *et al*., 2006; Stanton *et al*., 1994):

(2) Sv(C)=10log(C)+TS

where *C* is the net-catch estimate of each zooplankton group (ind. m^−3^). The theoretical volume backscatter at abundance level of 1 ind. m^−3^ was calculated, which is numerically identical to the TS and is referred to here as Sv(1). Of the species treated in the present study, the chaetognaths and the ctenophore *Mertensia ovum* are most likely the two taxa least suited to the bent fluid model used for calculating target strength (Stanton *et al*., 1998a,b). Neither possesses a hard exoskeleton or carapace like the other species (all crustaceans), and their target strengths are hence most likely overestimated.

From the theoretical estimate of backscatter for each zooplankton group, we are then able to identify those organisms that will dominate the measured acoustic signals. TS was calculated for each of the most common species from the region, with the lower limit in size being the older (≥CIV) copepodite stages of *Calanus* spp. at around 2.2 mm. For the Ice Station, with its general low abundance of zooplankton, we also took *Metridia longa* into consideration due to its relatively high abundance at this site. We used the TS value calculated for *Calanus* spp. as an approximation for *M. longa* as the two taxa resemble each other in external morphology and size. For the krill species, the size spectrum obtained during the cruise was used to sub-divide the population into two classes; large (mean length 18 mm) and small (mean length 8 mm) krill. All groups examined are hereafter referred to as “species”. To enable a viable comparison between each of the species' individual contributions to the measured backscatter levels, both the numerical difference (Δ Sv) in Sv(1) values between the species and the linear transformation (according to Stanton *et al*., 1994) of Δ Sv following Δ Sv = 10^(dB/10)^ are presented in Table III.

**Table III:** Calculation of the theoretical target strengths (TS) of the most abundant taxa based on measurements of organisms collected across all four stations, the relative difference (Δ) and its linear transformation (linear Δ).

Taxon/group	*L*	*s*	*D*	*β_D_*	TS	Sv (1)	Δ Sv	Linear Δ Sv
*Mertensia ovum*	0.018	0.8889	0.021	2.5	−74.0	−74.0	34.6	2884.0
*Themisto libellula*	0.014	0.214	0.003	4.611	−80.7	−80.7	27.9	616.6
Krill (large)	0.018	0.111	0.003	8.7	−85.0	−85.0	23.6	229.1
*Themisto abyssorum*	0.006	0.1667	0.001	3.187	−95.7	−95.7	12.9	19.5
Krill (small)	0.008	0.125	0.001	8.8	−100.0	−100.0	8.6	7.2
*Calanus* spp.	0.00226	0.24	0.00083	2.72	−102.1	−102.1	6.5	4.5
Chaetognaths	0.018	0.0277	0.001	18.7	−108.6	−108.6	–	1.0

*L*: mean body length in m, *s*: (standard deviation in *L*)/*L*, *D*: mean body width in m, *β_D_*: L/D, TS: target strength, calculated based on Eqn (1), Sv (1): theoretical backscatter of each taxon using a standard abundance of 1 ind. m^−3^ expressed as dB, Δ Sv: numerical difference in Sv (1) between taxa using the weakest scatterer as a reference expressed as dB, Linear Δ Sv: linear transformation of Δ Sv(1) using the equation Δ Sv = 10^(dB/10)^. Accordingly, one *T. libellula* has the same scattering potential as 616 Chaetognaths or 137 *Calanus* spp.

We then calculate a total estimated backscatter for each net haul, which we term the Sound Image (SI). The SI (using Equation 3) is the sum over all theoretical backscatter values for each species at each site and is based on the abundance data from the net hauls presented in Table II.

(3) $$\begin{array}{r} \text{SI}=10\;\log(10^{Sv_{1}/10}+10^{Sv_{2}/10}+\cdots +10^{Sv_{n}/10})\;\mathrm{dB} \end{array}$$

where Sv*_n_* is the estimated Sv value (Eq. 2) for species *n*.

The SI reflects the relative contribution (in dB) from each species to the total estimated backscatter, enabling a more direct comparison of backscatter contribution than from single species dB values alone.

The relative contribution to the SI of each species is presented as sectors within a pie chart, where the entire pie represents the entire SI value. All calculations for the SI are based on MIK nets (or WP3 at the Ice Station), except the values for calanoid copepods which are based on MPS samples in the equivalent depth intervals.

As the MIK net used to sample the macrozooplankton could not be closed, the SI value can only be determined unequivocally for the upper part of the water column at LSN and LM for each site. We make the assumption that zooplankton species performing DVM will have different abundances in the upper layer during LM compared with LSN due to DVM behaviour. Calculations for the theoretical Sv values also assume a homogenous distribution of scatterers, but by definition an SSL is an aggregation of organisms. Therefore, the calculated Sv values will under-estimate backscatter values compared with the measured backscatter within an SSL. Taking this into consideration, and the unavoidable problem of calibration (see above and Deines, 1999), the calculated theoretical Sv values are only comparable within each sample and location, and may only be used as an indication of relative change in backscatter between LSN and LM. As such, we restrict our analyses to the relative variations in backscatter values rather than attempting a direct comparison of absolute numbers between the measured and the theoretical backscatter values.

## RESULTS

The time series showing the absolute backscatter (Sv) distributions detected by the ADCPs at each site are presented in Fig. 2. In each case, the mean Sv value for the full deployment was calculated and only those backscatter values that exceeded the mean value are presented using a graduated scale where white represents the mean value. This allows only those regions of high backscatter to be shown, which represent the highest abundance of scatterers. A normalized measure of *in situ* ambient solar irradiance, E_0_ (PAR) from one fixed depth is overlain on the backscatter distribution. The right-hand panel in each sub-figure shows the mean Sv profile at LSN with the SSLs identified as the peaks in backscatter relative to the mean Sv value for the deployment. These SSLs are numbered for reference, with the circle indicating the position of maximum Sv value within each SSL (Fig. 2).

There is a difference in the number and vertical distribution of SSLs between each of the sample sites (Fig. 2). Focusing on the distribution of SSLs observed during LSN at each site (Table IV), we note the greatest level of complexity in terms of number of SSLs at LSN in Kongsfjorden (Fig. 2A). Here, we see four discrete SSLs at LSN, the three shallowest being typically 10 m in vertical extent while the deepest (SSL4) exceeds 100 m in extent but not associated with the seabed. These four SSLs are seen to ascend and coalesce during the period of sunset to form a single SSL at LM. In Rijpfjorden (Fig. 2B), there are two very clear SSLs at LSN, one rather diffuse and centred around 50 m (SSL1) and a deep one (SSL2) confined to the bottom 20 m. SSL2 makes a rapid migration to the surface during sunset, apparently merging with the shallower SSL1 to create a single dominant SSL in the upper 30 m at midnight. In Billefjorden, three SSLs can be identified at LSN; a rather weak layer at around 50 m (SSL1), a well-defined layer extending from around 115 to 150 m (SSL2) and a deep layer confined close to the seabed (SSL3). During sunset, all the SSLs are seen to ascend giving a strong SSL in the upper 25 m and a more diffuse SSL from the sea bed up to around 140 m. Finally, the Ice Station was characterized by a generally weak backscatter signal. Nevertheless, we identified two SSLs during LSN, one confined to the upper 20 m (SSL1) while the second is found below 250 m (SSL2). SSL2 is seen to ascend during sunset giving a single SSL at LM.

**Table IV:** Attribution of species to SSLs for each site reporting the depth range, temperature and salinity of each SSL.

Location/SSL	Depth range (m)	Salinity	Temperature (°C)	Ascent speed (mm s^−1^)	Suggested migrators
Kongsfjorden					
1	20–40	34.17	2.83	<6	*Mertensia ovum*
2	60–90	34.52	1.27	<6	*Calanus* sp.
3	120–140	34.57	0.94	<22	*Themisto abyssorum*/krill
4	150–280	34.78	2.13	<22	*Themisto abyssorum*/krill
Rijpfjorden					
1	30–60	34.06	2.72	<5	*Calanus* sp, Chaetognaths
2	150–bottom	34.55	−0.77	<22	Krill
Billefjorden					
1	30–50	33.86	1.00	<5	*Calanus* sp., Chaetognaths
2	120–140	34.62	−1.58	<10	Krill, *Th. libellula*
3	180–bottom	34.65	−1.53	<20	Hyperbenthos
Ice Station					
1	20–30	32.77	−1.38	<6	Calanoid copepods
2	250–	34.94	2.46	<20	*Themisto libellua*

The swimming speed is based on a simple calculation of the average speed of a SSL as it moves from depth (at LSN) to the surface.

**Fig. 3.**
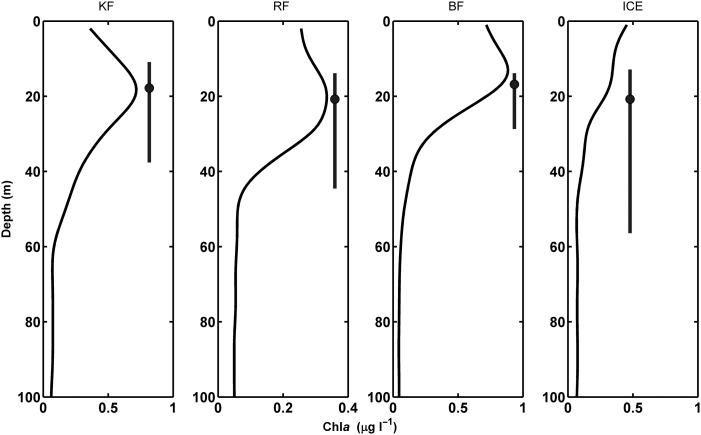
Fluorescence profiles measured as Chl *a* (μg l^−1^) at each site, Kongsfjorden (KF), Rijpfjorden (RF), Billefjorden (BF) and Ice Station (ICE). In each profile, the vertical extent of the surface SSL at LM is shown by a dark grey vertical line with a dot indicating the depth of the peak Sv within the SSL.

In Fig. 3, we show the relationship between the concentration of Chl *a* (μg L^−1^) in the upper 100 m and the position of the surface SSL at midnight (LM) at each of the four sites. The Chl *a* profiles are comparable at all three fjord localities with maximum values measured around 20. At each of these sites, the peak of the SSL at LM is also located at ∼20 m, coincident with the peak in Chl *a*. At the Ice Station, the surface SSL also shows peak backscatter at 20 m, but the highest Chl *a* is found immediately under the ice. In most sites, there appears to be a clear association between the vertical position of the zooplankton and the location of the Chl *a* maximum (Chl *a*_max_).

**Fig. 4.**
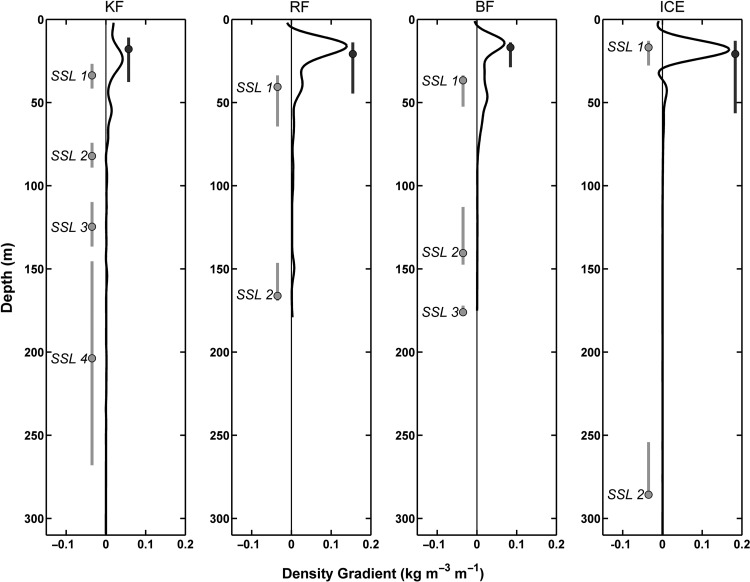
Profiles of the density gradient at each site, Kongsfjorden (KF), Rijpfjorden (RF), Billefjorden (BF) and Ice Station (ICE). SSLs identified during LSN (light grey vertical lines with dots indicating the depth of peak Sv) and LM (dark grey lines with dots at peak Sv).

**Fig. 5.**
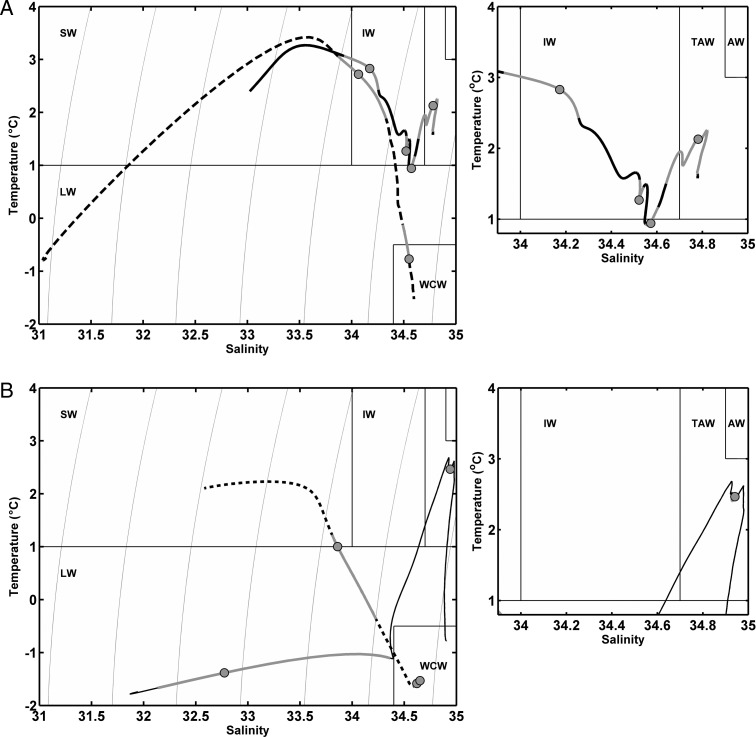
Temperature–salinity distributions in (**A**) Kongsfjorden (thick solid line) and Rijpfjorden (dashed line), (**B**) Billefjorden (dotted line) and Ice Station (thin black line). For each site, the occurrence of the SSLs at LSN is shown by the light grey portion of each line and with the dot indicating the depth of peak Sv within each SSL. Characterization of water masses follows Cottier *et al*. (Cottier *et al*., 2005) and Nilsen *et al*. (Nilsen *et al*., 2008): SW, Surface Water; LW, Local Water; WCW, Winter Cooled Water; IW, Intermediate Water; TAW, Transformed Atlantic Water; AW, Atlantic water. The left-hand figure shows the full temperature–salinity distribution while the right-hand subfigure highlights that part of the profile falling within the IW, TAW and AW domains. Thin grey lines in the left-hand figure are isopycnals ranging from 1025 to 1028 kg m^−3^.

The peak backscatter in the surface at LM is located precisely at the depth of maximum density gradient, the pycnocline (Fig. 4) with the SSL distributed through the lower part of the pycnocline at all sites. During daytime (LSN), the shallowest scattering layer (SSL1 at all sites) generally also occupied the lower part of the pycnocline, the Ice Station being the exception. Deeper SSLs were not associated with any significant density gradients. Rather, we see from Fig. 5 that LSN SSLs were located at inflections in the temperature–salinity curves. By definition, the inflection points correspond to layers in the water column bounded top and bottom by gradients in temperature and/or salinity. Consequently, an SSL located at an inflection point is within a layer of water that has well-defined temperature and salinity characteristics and which are distinct from any layers above and below. Water masses represent the broad definitions of these characteristic temperature and salinity relationships. Kongsfjorden shows the greatest complexity in its hydrographic structure (Fig. 5A), with multiple inflections in the temperature–salinity relationship indicative of distinct layers of water. The deep SSL4 is within the Transformed AW, being the warmest and most saline water in the fjord, while the intermediate depth SSL2 and SSL3 are located within interleaving layers of water. In Rijpfjorden, Fig. 5B, the deep SSL2 is in the coldest water mass of WCW which is a product of sea ice formation the previous winter (Nilsen *et al*., 2008). It is similar in Billefjorden, Fig. 5B, where the intermediate and deep SSLs are both within the WCW and therefore not separated by any gradients in the hydrography. At the Ice Station, the temperature–salinity distribution shows a classic form for the arctic halocline with cold and fresh SWs overlying the deeper, saline Atlantic layer (Rudels *et al*., 1996, 2005). We see that the deep SSL2 is located precisely at the warmest and most saline water mass (TAW) after a descent of over 250 m from the surface.

### The zooplankton community

The zooplankton community in the upper 50 m (100 m in Kongsfjorden) was sampled at all four sites during both LSN and LM (catch data summarized in Table II). Calanoid copepods were numerically the most dominant group at all sites, with *Calanus* spp. dominating within the fjords and *Metridia longa* at the Ice Station. The second most dominant group (numerically) was chaetognaths, followed by *Mertensia ovum*, krill and *Themisto* spp. (Table II). *Themisto libellula* were observed by divers (J. Berge, pers. Obs.) to be present at the Ice Station under the sea ice during daytime.

**Fig. 6.**
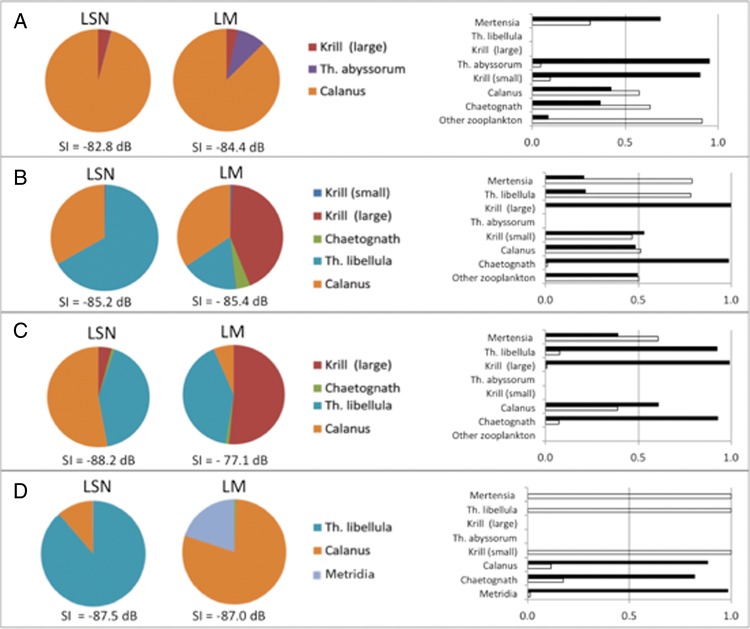
Sound Images and relative zooplankton abundance at LSN and LM for (**A**) Kongsfjorden (0–100 m), (**B**) Rijpfjorden (0–50 m), (**C**) Billefjorden (0–50 m) and (**D**) Ice Station (0–50 m). The Sound Images are represented as pie charts showing the relative contribution of each species to the theoretical backscatter value determined from catch data with the theoretical backscatter value given. Based on the likely overestimated TS value of *Mertensia ovum*, this species is not part of the SI calculation (see Discussion for details). The relative abundance of scatters at LSN (white bars) and LM (black bars) in the surface are given as a bar chart (right panels).

Lowest backscatter values were estimated for the chaetognaths and *Calanus* spp., highest for the ctenophore *Mertensia ovum*. The ratios of echo-energies (Δ Sv, dB) and their linear transformations are presented for all taxa examined in Table III. In terms of theoretical backscatter and the resulting SI in Fig. 6, *Calanus* spp. and *T. libellula* were the two most dominant taxa at all the sites, with the exception of krill during LM in Rijpfjorden and Billefjorden and *Metridia longa* during LM at the Ice Station. In Kongsfjorden (Fig. 6A), *Calanus* spp. dominated the SI both during LSN and LM, with *T. abyssorum* increasing in relative importance during LM. In both Rijpfjorden and Billefjorden (Fig. 6B and C), a comparable pattern of *Calanus* spp. and *T. libellula* dominating the SI during LSN occurred. At both these sites, the relative contribution of krill increased during LM, indicative of a strong krill DVM. At the Ice Station, where very few organisms were collected in the net samples, the SI during LSN was dominated by *T. libellula*, whereas the SI during LM was totally dominated by the calanoids (*M. longa* and *Calanus* spp.). Importantly, for all calculations of SI, the contribution by *Mertensia ovum* was disregarded, as this species is poorly suited to the bent fluid model used for calculating target strength (Stanton *et al*., 1998a,b) and would therefore dominate the calculation of theoretical backscatter.

Diel change in abundance of each taxon suggests that in Kongsfjorden (Fig. 6A, right panel) krill, *Mertensia ovum* and *T. abyssorum* were performing DVM in the upper 100 m. In Rijpfjorden (Fig. 6B, right panel), krill and chaetognaths show a diel displacement in abundance that is consistent with a DVM behaviour. In Billefjorden (Fig. 6C, right panel), all taxa except *Mertensia ovum* had a higher abundance in the upper 50 m during LM compared with LSN. At the Ice Station (Fig. 6D, right panel), *Metridia longa*, *Calanus* spp. and chaetognaths all had a higher abundance in the upper 50 m during LM than LSN.

## DISCUSSION

Our current understanding of DVM in the Arctic during day–night cycles as observed by acoustic techniques is characterized by a rather simple ascent/descent pattern of a single aggregation of zooplankton, or SSL (e.g. Berge *et al*., 2009; Cottier *et al*., 2006; Wallace *et al*., 2010). The distribution of SSLs recorded at LSN (Fig. 2) indicates that at all the locations in this study zooplankton migrate down to depth during daytime with a subsequent migration into the Chl *a*_max_ layer during the night. This pattern is consistent with classical ideas on DVM (see Hays, 2003 for a review). Also, all vertical migration events appear to be synchronized in time, which is a strong indication of a light mediated response. Nevertheless, the acoustic backscatter data from this study challenge the classical concept of simple DVM comprising a single migrating SSL. Instead, our data show a more complex DVM process with the formation and migration of multiple layers of zooplankton distributed through the water column at each study site. This evidence for complex patterns of DVM is the core result of this study.

### Sound Scattering Layers at LM (Fig. 3)

Our observations show that at all sites, there is a dominating SSL near the surface at LM, supporting the classical view of DVM behaviour. Importantly, the shallow SSL is formed from a coalescing of deeper SSLs that ascend to the surface at dusk. In Fig. 3, we show that the depth of the surface SSL is coincident with the region of enhanced Chl *a,* with the peak of backscatter typically coincident with the Chl *a*_max_. Colocation between the shallow SSL and the increased availability of food is a strong indication of the ascent of zooplankton in the DVM cycle being a biologically driven process (e.g. Ringelberg, 2010). The SSL and Chl *a*_max_ are found within the pycnocline (Fig. 4), a phenomenon that has been previously observed (Basedow *et al*., 2010; Dekshenieks *et al*, 2001; Holliday *et al*, 2010) and used for modelling the vertical migration of copepods (Wallace *et al*, 2013). Consequently, we interpret the consistent observation of surface SSLs at night as being zooplankton targeting the enhanced concentration of phytoplankton within the pycnocline.

### Sound Scattering Layers at LSN (Table IV and Fig. 4)

#### Shallow

The shallowest SSL at LSN was found just below the pycnocline having descended from the region of Chl *a*_max_ (Fig. 4). It is likely that chaetognaths were a key constituent of the surface SSL at those sites where they were found in high abundances, e.g. in Rijpfjorden and Billefjorden. DVM by chaetognaths is a known behaviour (Pearre, 1973; Sweatt, 1980) and the swimming speeds (Table IV) are appropriate. A candidate species in the surface SSL at Kongsfjorden was *M. ovum* which was generally found shallower than 100 m at similar densities during both LSN and LM. Moreover, it is reported to be restricted to the upper surface layer during autumn (e.g. Raskoff *et al*., 2005). At the Ice Station, the surface SSL was found precisely within the pycnocline and most likely dominated by *T. libellula* (SI, Fig. 6D). Interestingly, however, and based in particular on the net samples (Table IIB), the shallow layer during LM is composed also of *Metridia longa* and the ice-associated *Apherusa glacialis* (Berge *et al*., 2012b). Thus, the constant and slightly higher backscatter signal observed at the surface layer may have been the presence of ice fauna, moving out of and into the ice at different times of the day. *Apherusa glacialis* was found in nets only during night time, indicating movement away from the ice and into the pelagic zone under cover of darkness (Berge *et al*., 2012b).

Deep

Our data show that density gradients at the depth of the deepest SSLs are negligible. Rather, the depth of the deep SSLs at LSN shows a link to the water properties, particularly temperature. We note that in both Billefjorden and Rijpfjorden, the deepest SSLs migrated into the coldest water, WCW, while at the Ice Station and Kongsfjorden the deep SSL is located within the thermal maximum of AWs (Fig. 5A and B). We conclude that the vertical position of the SSLs represents a balance between predation risk, thermal optima and distance to the food-rich layers.

In terms of species composition (Table IV) of the deep SSLs, the relatively high ascent rates of the deep migrating SSLs suggest that krill and amphipods may be the main contributors. In both Rijpfjorden and Billefjorden, both groups exhibit a clear displacement in their abundance during LSN (Fig. 6B and C). Indeed, *Themisto* spp. and krill have previously been shown to perform DVM (e.g. Fortier *et al*., 2001; Kaartvedt, 2010) over long vertical distances (Berge and Nahrgang, 2013), and krill and *Themisto* spp. distributions and their contributions to the SI match the deep migrating SSLs at all four localities. We also saw evidence for deep SSLs undergoing partial ascent at midnight in both Rijpfjorden and Billefjorden (Fig. 2B and C).

The SIs for LSN and LM in Kongsfjorden are rather similar and differ only in the additional contribution from *Themisto abyssorum* at LM (Fig. 6A). Accordingly, one of the deep migrating layers in Kongsfjorden is most likely formed by the amphipod *T. abyssorum*. Of the taxa collected by the nets at the Ice Station, the chaetognaths and calanoid copepods both have vertical distributions in accordance with the migrating deep SSL (Table IIB). However, the abundances of calanoid copepods were high and they dominated the SI during LM (Fig. 6D). Further, the reported swimming speeds of chaetognaths (Hardy and Bainbridge, 1954) do not correspond with the ascent velocities observed for the SSL while the high swimming speed previously reported for *Metridia* spp. (up to 25 mm s^−1^, Miller, 2004) is within the observed range SSL (Table IV). Importantly, however, the lower limit of the ADCP in our study was around 300 m, and the SSL probably started its ascent from below this depth. Hence, conclusions concerning the ascent rate and absolute depth distributions of the SSL during LSN are incomplete.

Intermediate

Kongsfjorden and Billefjorden both show SSLs during LSN at intermediate depths (Figs 2 and 4). In Kongsfjorden, the surface SSL1 and the intermediate SSL2 could both be assigned to calanoid copepods which showed high abundance and theoretical backscatter within the upper 100 m during LSN and LM. Further, given that *Calanus* spp. dominated the SI at both LM and LSN (Fig. 6A), indicative of migration being restricted to the upper layers, and the reported swimming speed of *C. finmarchicus* of 4.2–18.3 mm s^−1^ (Mauchline, 1998), the intermediate SSL2 in Kongsfjorden (Fig. 4, Table IV) is likely to be composed of calanoid copepods. Calanoid copepods have been shown to constitute up to 80% of the total mesozooplankton biomass in Arctic region (Søreide *et al*., 2008), and they have also been suggested as candidates for acoustically detected DVM patterns from two of the four study sites used herein (Berge *et al*., 2009; Cottier *et al*., 2006). In Billefjorden (Grigor *et al*., 2014), it is likely that the intermediate SSL2 is composed of taxa migrating from the surface (krill and *T. libellula*, Table IV) being distinct from the deep SSL3 associated with deep migrating hyperbenthic taxa. Despite the rigorous sampling effort around both LSN and LM, the migratory species within these SSLs cannot be identified unequivocally as the sampling protocols were designed with the expectation of single rather than multiple SSLs.

### Environmental factors driving complex DVM

DVM is a ubiquitous feature of mid-latitude oceans whereby the zooplankton population resides at depth during daylight hours and moves *en masse* towards the surface during darkness (e.g. Hays, 2003; Fortier *et al.*, 2001). While the proximate cue for such behaviour is widely accepted to be changes in illumination, specifically the absolute and relative light intensity (e.g. Fortier *et al.*, 2001; Ringelberg and Van Gool, 2003; Tarling *et al*., 2002), the vertical extent of migration is often interpreted in terms of biological processes; mainly relating to community composition, predation and feeding (Fortier *et al*., 2001; Ringelberg, 2010; Tarling *et al*., 2014). Our result concur with these general features in that we observe a strong vertical displacement of biomass that is correlated with the diel change in solar illumination across all four examined stations. Importantly however, although the ascent/descent may be initiated by light, the final “resting” depth appears to be determined by hydrography rather than light. We suggest that this is the result of a trade-off between predator avoidance and a thermal optimum for particular species. Therefore, a site with rather complex hydrography is likely to see a more complex DVM pattern. This is supported by the fact that the number of SSLs at each site varies, while the zooplankton community composition remains quite similar. Indeed, the site with the fewest number of different scatterers (Table II, Fig. 6), Kongsfjorden, is also the site with the most complex hydrography and shows the highest number of SSLs.

Other investigators have noted the relationship between zooplankton community structure and the local hydrography (e.g. Schulz *et al*., 2012; Willis *et al*., 2006, 2011 for some recent examples). Lawson *et al*. (Lawson *et al*., 2004) related the vertical positioning during DVM of macrozooplankton (krill) to the occurrence of distinct water masses. However, that study regarded the vertical migrating community as a single entity, not a complex community segregated into several SSLs that might display affinities towards different water masses (see also Ashjian *et al*., 2008). Laboratory experiments investigating DVM in estuarine systems have shown that zooplankton will adjust their vertical position or migratory behaviour in response to the presence of haloclines (Lougee *et al*., 2002). The explanation for this has been proposed to be one of energetics, with less energy required to remain suspended in more saline dense water leading to accumulations of organisms in and below the halocline (Lougee *et al*., 2002). Similar separation of species has been observed in relation to the thermocline, with *C. finmarchicus* occupying colder water below and C*. helgolandicus* in the warmer fresher water above a thermocline (Williams, 1985). Other experimental investigations have determined that the vertical distribution of zooplankton is in response to density rather than salinity (Harder, 1968).

Our observations (Figs 4 and 5) challenge the importance of density as a driver of vertical structure (Harder, 1968), with multiple SSLs located in regions of uniform density in at least two of the fjords (Kongsfjorden and Billefjorden, Fig. 4). However, our data show that the hydrographic structure of the water column does appear to correlate with both the number and the position of SSLs. In Kongsfjorden, with the highest number of SSLs, the three deepest SSLs are associated with distinct temperature and salinity characteristics (Fig. 5A). Precise vertical positioning is seen in Rijpfjorden where the deep SSL matches the upper boundary of the WCW layer, and at the Ice Station where the deep SSL matches the thermal maximum of the AW layer (Fig. 5A and B, respectively). Another indication for a physical forcing of the DVM patterns comes from a comparison between the data from Kongsfjorden in 2002 (Cottier *et al*., 2006) and 2010 (data herein). Both data sets were collected using ADCPs of the same frequency at the same site and time of year. While the data from 2002 showed only one vertically migrating SSL, the number had increased to four in 2010. During this transition period, the macrozooplankton species present in the fjord remained relatively stable (Hop *et al*., 2002; Kwasniewski *et al*., 2012), although it should be noted that there has been a conspicuous shift in the relative abundance and dominance of these species (Kwasniewski *et al*., 2012). The hydrography, however, has changed dramatically since 2002, with pronounced and episodic influx of AW as well as ice-free conditions since 2006 (Berge *et al*., 2005; Willis *et al*., 2008; Wallace *et al*., 2010, pers. Obs.), supporting the hypothesis that a physical forcing of DVM patterns is more important than previously assumed (e.g. Klevjer *et al*., 2012).

A potentially important environmental factor behind the observed DVM patterns is the differences in predator distributions between the study sites; as also suggested in a comparative study of DVM patterns in Kongsfjorden and Rijpfjorden (Wallace *et al*., 2010). A number of studies have shown that the presence of specific predators can cause rapid behavioural responses among prey (Bollens and Frost, 1989a,b,^1991^; Fortier *et al*., 2001; Tarling *et al*., 2002). Important predators such as the polar cod (*Boregadus saida*) or little auks (*Alle alle*) are known to be common at all of the study sites (Falk-Petersen *et al*., 2008; Renaud *et al*., 2012), and might be an important mediator of the distribution of zooplankton. Other and more Atlantic species such as the Atlantic cod (*Gadus morhua*) or capelin (*Mallotus villosus*) are more dominant in Kongsfjorden compared with the other study sites (Berge *et al*., in press; Nahrgang *et al*., 2014; Renaud *et al*., 2012). Although not observed in this study, Ohman (Ohman, 1990) showed a reversed DVM of herbivores in the presence of both tactile and visual predators. However, if visually searching predators such as the polar cod or capelin exert a high predation risk, one would expect size-dependent migrations in the prey, with larger individuals (most easily spotted by the predator) migrating to greater depth than smaller prey (Hays, 2003). Such a size-dependent migration is partly seen in our study, with the larger macrozooplankton constituting the deeper SSLs, although it is impossible to isolate a potential predator avoidance mechanism from merely a swimming-speed dependent segregation of migrating zooplankton. Other potential predatory species known to be important in the system include *Themisto* spp. (Kraft *et al*., 2013, this study) and chaetognaths (Falkenhaug, 1991) which are known to feed on calanoid copepods (Falkenhaug, 1991).

### Characteristics of SSLs and conclusion

We present evidence for the occurrence of distinctly different patterns of DVM between sites that are relatively close geographically with a zooplankton community comprising the same set of species (Fig. 6), but with contrasting hydrographic characteristics. *Calanus* spp. has previously been identified as a major contributor to the vertically migrating component of the zooplankton community during late summer and autumn (Blachowiak-Samolyk *et al*., 2006; Cottier *et al*., 2006; Falk-Petersen *et al*., 2008). We, however, based upon our comparison between acoustic and net data conclude that the acoustic backscatter from calanoid copepods is typically overwhelmed by the signal from the larger zooplankton species, krill and *Themisto* spp. in particular, when both are present in the water column. Also, we argue that the differences in the hydrographic structures, particularly the local temperature characteristics can give rise to complexity in DVM patterns. Based on the distribution of SSLs in Kongsfjorden and the Ice Station, organisms do not appear to be migrating down to colder water in which a lowered metabolism might optimize an energy budget (Ikeda, 1985). The deepest migrating SSLs at both of these stations migrate down through colder water masses to temperature maxima. Similarly, the vertical positioning of the SSLs seems only weakly correlated with salinity, although all of the deeper SSLs are associated with salinity levels above 34.50 PSU.

Our study has revealed a complexity of polar marine systems that has been poorly acknowledged. First, we have documented the existence of multiple SSL in regions previously believed to be characterized by simple, uniform patterns of DVM (e.g. Berge *et al*., 2009; Cottier *et al*., 2006; Wallace *et al*., 2010). Second, we document the distinctly different patterns observed across the sites examined (Figs 2–5), indicating that autumn DVM in the Arctic comprises a complex behaviour by a wide variety of planktonic taxa. The different taxa performing DVM are likely to have distinct feeding preferences, migration-depth patterns and faecal pellet properties.

As there appears to be a positive correlation between complexity of hydrography and number of SSLs, any long-term change in Arctic hydrography may effectively lead to marked changes in both behaviours of individual species as well as in overall ecosystem function. There are examples of on-going changes linked with e.g. the amount of inflowing AW, alterations of freshwater input and local mixing of water masses, potentially leading to changes in pelagic complexity and community segregation as different scatterers position themselves at different depths throughout the water column. Ultimately, this suggests that while DVM of Arctic zooplankton could have significant implications on retention and export of organic and inorganic compounds through the biological carbon pump on Arctic shelves, a more thorough insight into which species are performing the DVM is needed in order to fully understand their impact.

## SUPPLEMENTARY DATA

Supplementary data can be found online at http://plankt.oxfordjournals.org.

## FUNDING

This work is a contribution to the NFR funded project CircA; Circadian rhythms of Arctic zooplankton from polar twilight to polar night—patterns, processes and ecosystem implications (project number 214271/F20). It is also a contribution to the Natural Environment Research Council program Oceans 2025. Funding to pay the Open Access publication charges for this article was provided by the CircA project.

## Supplementary Material

Supplementary Data
