# Supplementary material for: Arctic complexity: a case study on diel vertical migration of zooplankton
Source: J Plankton Res. 2014 Jul 9;36(5):1279–97. doi: 10.1093/plankt/fbu059 (PMC4161229; doi:10.1093/plankt/fbu059)
Supplement: Supplementary Data [file supp_36_5_1279__index.html]

Supplementary Data 

# Arctic complexity: a case study on diel vertical migration of zooplankton

## Supplementary Data

Supplementary Data

**Files in this Data Supplement:**

- Supplementary Data - Docx file
