# Supplementary material for: Arctic complexity: a case study on diel vertical migration of zooplankton
Source: J Plankton Res. 2014 Jul 9;36(5):1279–97. doi: 10.1093/plankt/fbu059 (PMC4161229; doi:10.1093/plankt/fbu059)

Supplementary Information Fig. S1: Distribution of absolute backscatter (Sv in dB) observed over a 36 hour period for the four sampling sites (A) Kongsfjorden, (B) Rijpfjorden, (C) Billefjorden and (D) Ice Station. The scale (colour) bar is the same for all sites and covers the entire range of backscatter values measured during the ADCP deployments. All other aspects of the figure are as per Fig. 2.

Supplementary Information Fig. S2: Krill (*Thysanoessa* sp) photographed *in situ* in Kongsfjorden.


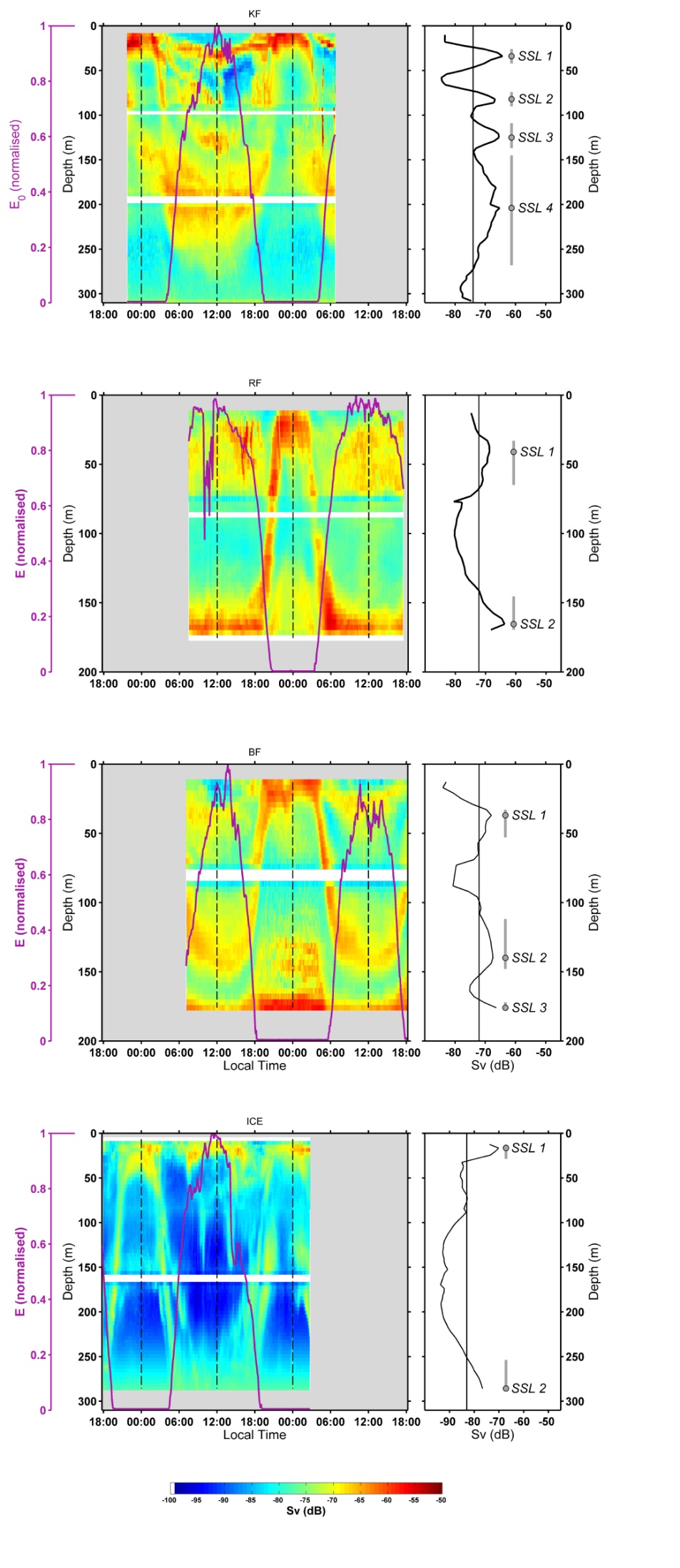


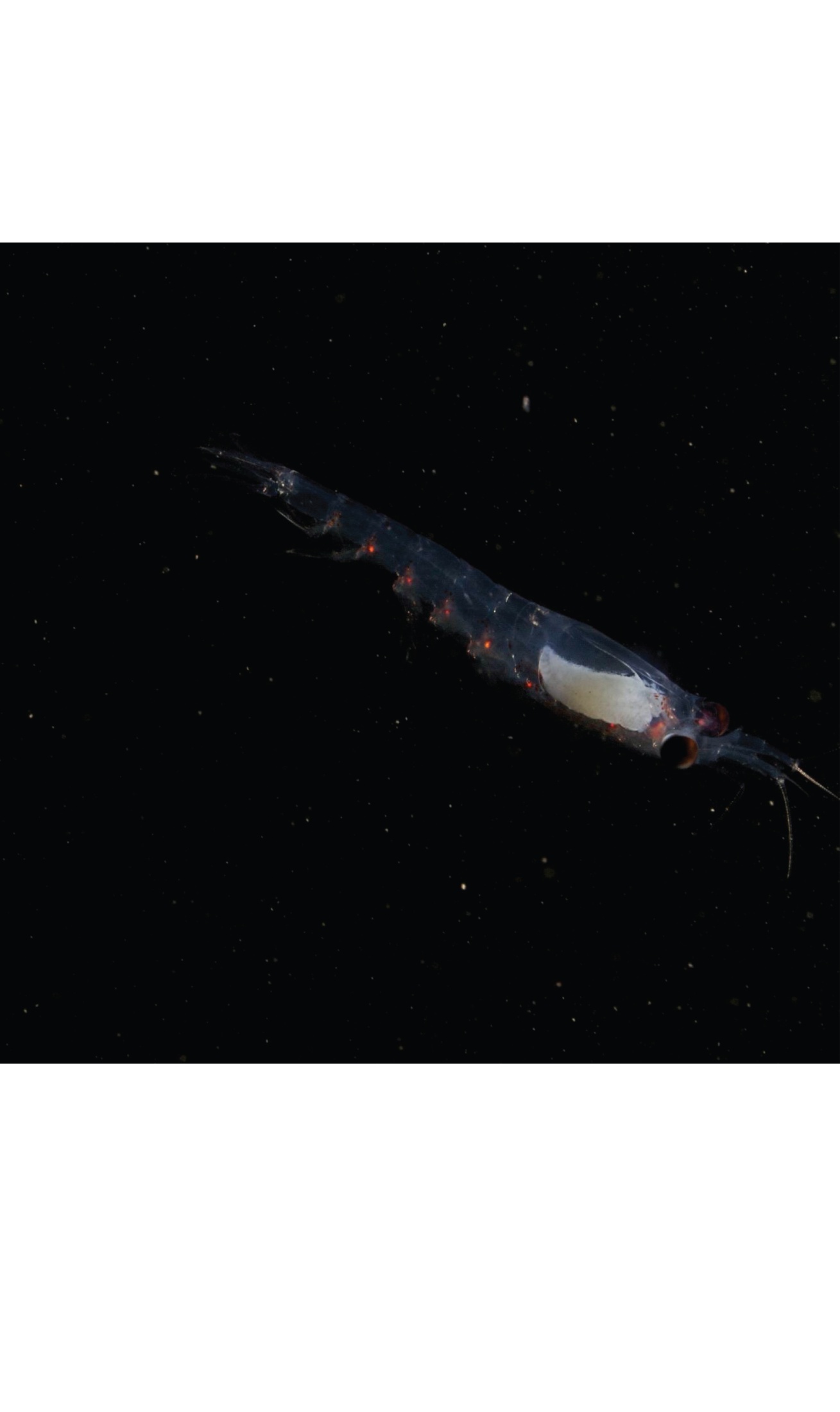

Supplement: Supplementary Data [file supp_fbu059_fbu059supp.docx]
